# Psychosocial Interventions for Alcohol Use Among Problem Drug Users: Protocol for a Feasibility Study in Primary Care

**DOI:** 10.2196/resprot.2678

**Published:** 2013-08-02

**Authors:** Jan Klimas, Rolande Anderson, Margaret Bourke, Gerard Bury, Catherine Anne Field, Eileen Kaner, Rory Keane, Eamon Keenan, David Meagher, Brian Murphy, Clodagh SM O'Gorman, Thomas P O'Toole, Jean Saunders, Bobby P Smyth, Colum Dunne, Walter Cullen

**Affiliations:** ^1^Centre for Interventions in Infection, Immunity and Inflammation (4i) and Graduate Entry Medical SchoolFaculty of Education & Health SciencesUniversity of LimerickLimerickIreland; ^2^UCD School of Medicine and Medical ScienceCoombe Healthcare CentreDublinIreland; ^3^The Charlemont ClinicDublinIreland; ^4^Addiction Treatment Services, South Western Area Health BoardDublinIreland; ^5^Institute of Health and SocietyMedical FacultyUniversity of Newcastle upon TyneNewcastle Upon TyneUnited Kingdom; ^6^HSE Mid-WestRegional Drug Co-ordination UnitLimerickIreland; ^7^Addiction Services HSE Dublin Mid LeinsterDublinIreland; ^8^HSE Primary CareGalwayIreland; ^9^Warren Alpert Medical School of Brown UniversityProvidence, RIUnited States; ^10^CSTAR CentreUniversity of LimerickLimerickIreland; ^11^Department of Public Health and Primary CareTrinity College DublinDublinIreland

**Keywords:** complex intervention, screening, brief intervention, alcohol, methadone maintenance, primary health care, general practice, substance-related disorders

## Abstract

**Background:**

Alcohol use is an important issue among problem drug users. Although screening and brief intervention (SBI) are effective in reducing problem alcohol use in primary care, no research has examined this issue among problem drug users.

**Objective:**

The objective of this study is to determine if a complex intervention including SBI for problem alcohol use among problem drug users is feasible and acceptable in practice. This study also aims to evaluate the effectiveness of the intervention in reducing the proportion of patients with problem alcohol use.

**Methods:**

Psychosocial intervention for alcohol use among problem drug users (PINTA) is a pilot feasibility study of a complex intervention comprising SBI for problem alcohol use among problem drug users with cluster randomization at the level of general practice, integrated qualitative process evaluation, and involving general practices in two socioeconomically deprived regions. Practices (N=16) will be eligible to participate if they are registered to prescribe methadone and/or at least 10 patients of the practice are currently receiving addiction treatment. Patient must meet the following inclusion criteria to participate in this study: 18 years of age or older, receiving addiction treatment/care (eg, methadone), or known to be a problem drug user. This study is based on a complex intervention supporting SBI for problem alcohol use among problem drug users (experimental group) compared to an “assessment-only” control group. Control practices will be provided with a delayed intervention after follow-up. Primary outcomes of the study are feasibility and acceptability of the intervention to patients and practitioners. Secondary outcome includes the effectiveness of the intervention on care process (documented rates of SBI) and outcome (proportion of patients with problem alcohol use at the follow-up). A stratified random sampling method will be used to select general practices based on the level of training for providing addiction-related care and geographical area. In this study, general practitioners and practice staff, researchers, and trainers will not be blinded to treatment, but patients and remote randomizers will be unaware of the treatment.

**Results:**

This study is ongoing and a protocol system is being developed for the study. This study may inform future research among the high-risk population of problem drug users by providing initial indications as to whether psychosocial interventions for problem alcohol use are feasible, acceptable, and also effective among problem drug users attending primary care.

**Conclusions:**

This is the first study to examine the feasibility and acceptability of complex intervention in primary care to enhance alcohol SBI among problem drug users. Results of this study will inform future research among this high-risk population and guide policy and service development locally and internationally.

## Introduction

### Overview

Problem alcohol use is associated with adverse health and economic outcomes, all the more so among problem drug users (eg, individuals currently using illicit drugs or trying to abstain from other illicit drugs such as benzodiazepines, cocaine, or heroin) [1,2]. Such alcohol use may decrease in response to psychosocial interventions whose benefits have been demonstrated in general adult populations. For example, a comprehensive review by Raistrick et al presented data on the effectiveness of many such interventions, including screening, further assessment, brief interventions, and alcohol-focused specialist treatment [3].

Primary care may have an important role in addressing problem alcohol use among problem drug users. Its potential impact on screening for alcohol problems and providing appropriate interventions in the general population has been described [4], although a recently published randomized trial indicates that more intensive primary-care-based interventions provide little by way of additional benefit to patient information alone [5]. Internationally, screening and brief interventions (SBI) are recommended as a treatment of choice for reducing alcohol use among problem drinkers in primary care [6,7], but these have not been tested in people who are addicted to other substances and who attend primary care [8]. It is important to address this issue because of the serious complications associated with problem alcohol use in this population, that is, the potential to increase the likelihood of a relapse to problem drug use, medical/psychological complications, liver disease, and so on [1,2].

Similar to other evidence-based interventions, the evidence on SBIs translates slowly into practice [9-11], and the findings from implementation studies are contradictory. For example, while a systematic review of interventions focused on increasing the use of SBI for hazardous alcohol consumption in primary care recommended complex, multicomponent strategies [12], a recent trial concluded that such a “tailored, multifaceted program aimed at improving general practitioner (GP) management of alcohol consumption” failed to show an effect and proved difficult to implement [13]. This also contradicts the conclusions of a recent paper, “real world evidence supports theory” of SBIs [14].

More impetus to this contradictory debate has been added by recent implementation studies and a controlled trial among problem drug users in secondary care that demonstrated feasibility of implementing SBIs among problem drug users in secondary care but suggested a controlled pilot study was necessary to establish key parameters for a similar evaluation in primary care [15-17]. The present study is designed to evaluate these issues.

### Previous Work in Ireland and Its Relation to Complex Intervention Theory

This protocol builds on our ongoing program of research that indicates (opiate) addiction treatment should also incorporate interventions that address problem use of alcohol and other illicit substances. For example, a national cross-sectional study reported that 35% of 196 patients attending GPs for methadone treatment also had problem alcohol use [18], while findings from a subsequent qualitative study highlight the need for a complex intervention to address this problem in primary care [19].

The UK Medical Research Council (MRC)’s “Framework for the Development and Evaluation of Complex Interventions for Randomized Controlled Trials (RCTs)” [20], which suggests following core phases to the development of complex health service interventions, informed the development of the intervention under study.

#### Preclinical Phase: Theory and Problem Identification

A national prevalence study showed problem alcohol use among patients attending general practice for methadone maintenance was high (35%) [18]. A review of scientific evidence found no studies examining this issue in primary care, but research in secondary or community care settings suggests that this type of intervention can be effective among problem drug users [21].

#### Phase 1: Modeling

The development of the complex intervention and clinical guidelines is informed by Cochrane Systematic review, qualitative interviews with health care providers and patients, and clinical guidelines.

Cochrane Systematic review was used to assess “psychosocial interventions for problem alcohol use in illicit drug users” [8]. Qualitative interviews with health care providers and patients that showed that barriers to implementation of alcohol intervention for drug users in primary care include patient factors, health care professional factors, and structural issues. The implementation strategies should utilize educational and support systems [19]. Clinical guidelines—informed by the findings of qualitative interviews, expert opinion through a Delphi-facilitated expert consensus process, and a Cochrane Systematic Review [8]—advocate SBI for problem alcohol use among problem drug users.

#### Phase 2: Exploratory Study

A pilot study in addiction clinics showed that SBIs are effective in reducing alcohol consumption among opiate-dependent patients [16]. There is a current proposal to establish the acceptability and effectiveness of the intervention by conducting a feasibility study in primary care.

This protocol reflects the development and piloting phases of the MRC’s “Framework for the design and evaluation of complex interventions to improve health” [20,22]. The present study will provide key parameters regarding the feasibility and acceptability of the intervention to patients and practitioners. As such, this research is essential to inform the design and conduct of a larger cluster randomized controlled trial (RCT).

The specific objectives of the study are as follows. First, this study aims to develop a complex intervention that will enhance SBI for problem alcohol use among problem drug users in primary care. This study will help to establish the practical feasibility and acceptability of complex intervention (1) by conducting a pilot study (with randomization at the level of practice), (2) exploring the feasibility and acceptability of the intervention under study and related research procedures to GPs, practice nurses, and patients, and (3) exploring the fidelity of the interventions as delivered in practice. Finally, we can decide to inform the subsequent design of a definitive cluster RCT by describing the optimum configuration of the complex intervention and by estimating the key parameters in such a trial (ie, practice/patient recruitment and retention rates, intraclass correlation coefficient for primary outcome measures, and the likely effect of intervention under study on these measures).

## Methods

### Overview of Study Design

Psychosocial intervention for alcohol use among problem drug users (PINTA) is a pilot feasibility study of a complex intervention to promote SBI for problem alcohol use among problem drug users, with cluster randomization at the level of general practice, and integrated qualitative process evaluation, involving general practice in two regions.

### Study Population

#### Recruitment and Random Selection of Practices

The following practices will be invited to participate, given written information on the study and their interest in participating:

- All practices in two regions—Health Services Executive (HSE) Midwest and Dublin Mid-Leinster regions
- All practices that have been involved in previous related research with our group [18,23-29]
- General practices in the study regions that are affiliated with two of the Ireland’s six medical schools [30,31]

Practices will be eligible to participate if they are registered to prescribe methadone and/or have at least 10 patients currently receiving addiction-related care.

Of those who confirm their *interest* in the study and who are eligible to participate, a stratified random sampling technique will be used to select 16 practices.

Sampled GPs will be contacted about their participation, given further information on the study (eg, what their involvement will entail) and consulted about patient recruitment. The research team will telephone those not replying. Each practice will be visited by the principal investigator or lead researcher and provided with the information about the research program.

To ensure comparability between intervention and control groups for key practice characteristics, a restricted allocation involving stratified approach to randomization will be adopted. Prior to randomization, GPs who express their interest in participating will be grouped according to the level of training in providing addiction-related care (level 1 and 2), geographical location (Dublin/Midwest), with 16 randomly selected GPs using an independent remote randomization service.

To prescribe methadone, GPs are subject to clinical audit and must complete special training, while GPs providing methadone treatment for 15 or more patients are subject to more regular audit and advanced training. GPs who prescribe methadone for less than 15 patients are referred to as “level 1 GPs,” and those prescribing for 15 or more as “level 2 GPs.” Initiation of methadone therapy, treatment of patients with more complex medical and psychosocial needs (including alcohol dependence), and unstable drug use are only permitted by specialist addiction treatment services or by “level 2 GPs.” A more complex, difficult cohort of patients is attended by level 2 GPs and this might have implications for the success of the intervention. Therefore, it will be introduced in the data analysis as a potential confounder.

#### Identification and Recruitment of Patients

Before introducing the complex intervention, each participating practice will engage in an intensive, 2-week period of patient recruitment, an approach we found most effective in previous qualitative work with this population [19]. This 2-week period will be supported by a member of the research team and will aim to: (1) establish a “disease” register of patients, (2) obtain contact details for and informed consent from eligible patients, (3) review the clinical records of patients who consent to participate in the study, and (4) collect baseline data, including patient demographics and current care process/outcome measures from clinical records.

Patients will be eligible to participate if they are 18 years of age or older, receiving addiction treatment/care (eg, methadone), or known problem drug user, and attending a participating general practice for general medical care. They will be excluded from the study if they have language difficulties (ie, unable to speak, read, and write English well enough to complete study questionnaires), are acutely intoxicated, and/or are cognitively impaired (including severe mental health illness) to the extent that they are unable to provide informed consent to participate.

Systematic random sampling of patients in participating practices is difficult in studies among this population [25]. Hence, a standardized nonprobability sampling framework will be used to identify *consecutive* patients from each practice on whom data will be collected for the purpose of the study. Potential patient selection bias will be assessed in the exploratory data analysis, by comparing the sociodemographics of the included patients with all patients, who were identified as problem drug users, in each practice.

Patients who consult a GP taking part in the study, and who in the clinical opinion of the GP are eligible to participate in the study (see inclusion criteria above), will be given written information on the study. Those interested in participating will be invited to meet a researcher who will be at the practice during the recruitment period. At this meeting, interested patients will be given further information on the study and will have an opportunity to ask questions from the researcher. If patients consent to participate, they will be asked to sign a consent form and complete a self-/interviewer-administered questionnaire that includes problem alcohol use and other outcome measures, if necessary with the assistance of the researcher at T1 (ie, Time 1, at baseline) and T2 (ie, Time 2, at 3 months follow-up). This applies to patients in both the intervention and control groups.

**Figure 1:**
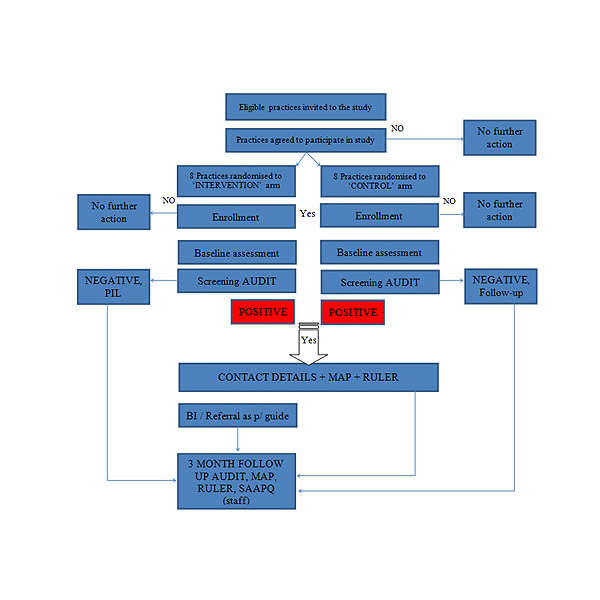
CONSORT diagram: participant flow and follow-up.

Following completion of the self-/interviewer-administered questionnaire with the researcher, patients in the intervention practices will be screened for problem alcohol use and delivered the brief intervention by their GP/practice team (at their earliest convenience). Patients in the control arm will receive the “Less is more” leaflet (a guide to rethinking your drinking, HSE, 2008) from the researcher. A “thank you” letter will be sent to all GPs and patients within 2 weeks of receiving completed study instruments/intervention. A reminder letter will be sent to all GPs and patients 5 weeks before the follow-up assessments, informing them of the anticipated time/date of their appraisal. Figure 1 presents the CONSORT diagram of participant flow and follow-up.

#### Power Calculations and Sample Size Estimates

The goals of this study are to examine the feasibility, acceptability, and effectiveness of the complex intervention. As observed in previous studies, with respect to the feasibility component, the present study aims to achieve 20% recruitment/consent rate (ie, number of invited GPs who confirm their interest in the study) [19], 75% participation rate (ie, the number of participants allocated to the intervention arm who will receive/complete SBI) [5], and 75% retention or follow-up rate [5].

Based on the recommendations for good practice in pilot studies [32,33], we estimate that 160 patients (attending 16 general practices) will be adequate to calculate the actual recruitment and retention rates (ie, *feasibility*) for a sample of patients recruited in primary care and provide data on *acceptability* of study processes and outcome measures, which will inform a future definitive trial. This pilot study is not powered to determine effectiveness of SBI on reduction of alcohol consumption among problem drug users. The proportion of patients who reduce their alcohol consumption will be used to predict the sample size and length of follow-up for a future definitive RCT.

### Intervention

#### Overview

A staggered intervention design will be adopted, whereby participating practices randomized to the intervention arm of the study will be provided with the complex intervention for the duration of the study period, while practices randomized to the control arm of the study will provide usual care to patients for the duration of the study and will be provided with the complex intervention thereafter (ie, delayed intervention). Such an approach was used successfully in our previous cluster randomized controlled study to improve screening for hepatitis C among problem drug users attending general practice in Ireland [25].

#### Control Intervention

All practices (control and intervention arms) will be required to establish a “disease” register of “problem drug users” before the study onset. They will identify potential participants and recruit them for the study. At this stage, participants will be asked to sign an informed consent form. The research team will conduct interviews (telephonic or in person) to determine problem alcohol and other drug use and demographic details at baseline and at 3 months follow-up. Researchers will facilitate data collection (including morbidity and primary/secondary care utilization) from clinical records. Participating practices will be offered €50 per patient recruited to study upon receipt of completed data [34].

We consider the above engagement with practices as close to “usual care” as possible while still allowing evaluation of the complex intervention. To enable the development of a practice register of people with problem drug use, clinical records and prescribing information will be reviewed. For practices who use electronic patient records, an International Classification of Primary Care disease code (P19) will be assigned to patients who meet the criteria of European Monitoring Center for Drugs and Drug Addiction for problem drug use. For practices who use paper records, this register will be developed in a hard copy.

#### Experimental Intervention

A complex intervention will be delivered to practices assigned to the intervention arm at two levels: (1) practice level and (2) patient level.

Interventions that are delivered at practice level include CME/CPD-accredited education delivered both internally (practice-based academic detailing) and externally (seminar), dissemination of clinical guidelines, other resources to facilitate implementation at practice level (eg, contact details/referral information for local services).

All practices will participate in the external education (seminar). Internal education (practice-based academic detailing) will be offered on as-needed basis, depending on practice resources and experience with SBI [35]. Academic detailing and support will be available to practices during the 3 months study period. The number and duration of these visits will be used to predict the level of support for a future definitive RCT.

At patient level, SBI (10-15 minutes) is delivered to patients.

### Data Collection

#### Timeline

At baseline, demographic details and data on primary/secondary outcome measures will be collected by reviewing clinical records and by patients completing study instruments. At follow-up, data will again be collected by reviewing clinical records and by patients completing study instruments. Participants will be invited to complete a follow-up interview with a researcher to include primary/secondary outcome measures. A purposive sample of patients in the “intervention” arm will be interviewed regarding their experience in care for alcohol-related problem in the preceding 3 months.

Quantitative data will be collected at baseline (T1) and at 3 months follow-up (T2) using clinical records (T1, T2), self-/interviewer-administered questionnaires and semistructured interviews (patients, T1, T2), and self-administered questionnaires, including open-ended questions (practitioners, T2).

#### Outcome Measures

Table 1 summarizes the key data being collected during the study.

##### Staff and Organization Measures

Health care professionals at participating practices will be asked to complete a self-administered questionnaire that will elicit data on practice/professional details, experience of training, intervention fidelity (The NIH “Behavior Change Framework” [36]), and Shortened Alcohol and Alcohol Problems Perceptions Questionnaire (SAAPPQ).

##### System Measures

System measures at the examination include the total number of patients screened for alcohol problems (and method of screening), the number of positive screening, and the number of patients receiving any alcohol intervention (including referral). Results of chemical tests for alcohol and drugs (eg, breathalyzer or urine tests) conducted by GPs will be also retrieved using the practice records (T2) to verify self-report measures.

##### Patient Measures

These measures include indirect examination and direct examination. At baseline and follow-up, the study battery will include the following: (1) Alcohol Use Disorders Identification Test (10 items) (AUDIT), (2) Maudsley Addiction Profile (MAP), and (3) Readiness Ruler.

AUDIT developed by the World Health Organization is used to identify a continuum of problem alcohol use [21,38].

MAP is a brief, structured questionnaire for treatment outcome research and measures problems specifically in four areas: substance use, health risk behavior, physical and psychological health, and personal/social functioning [18,39].

Readiness Ruler will assess patient’s motivational state regarding changing their drinking behavior [41].

##### Financial Incentives

Participating practices will be offered €50 per patient to compensate for the extra administration work as in a similar trial [34]. We consider this a conservative level of remuneration given the additional work involved for participating practices [42].

**Table 1:** Primary and secondary outcome measures to be used at the baseline and/or follow-up examinations.

Aim/Target group	Patient measures	Staff and organization measures	System measures
Feasibility	Indirect (review of clinical records): Sociodemographic characteristics and general medical morbidity (ie, clinical records review using a structured instrument developed previously [24]) at baseline	Self-administered baseline questionnaire to include: Practice/professional details Experience of training Adherence to intervention guide/manual assessed with the NIH “Behavior Change Framework” [36] (includes five intervention adherence strategies: intervention design, training procedures, delivery of intervention, receipt of intervention, and enactment of SBI skills) at follow-up Shortened Alcohol and Alcohol Problems Perception Questionnaire (SAAPPQ)	Indirect (review of clinical records): Current and previous practice, with regards to screening and intervention for problem alcohol use among identified problem drug users Numbers of patients who were (1) screened for alcohol, (2) offered a brief intervention, (3) received the brief intervention, and (4) referred to a specialist at follow-up
Acceptability	Patients’ experience of intervention: semistructured interviews at follow-up (via telephone or in person)	Postal survey to include: SAAPPQ [37] at baseline and follow-up Health care professionals’ experience of the intervention: free text in questionnaires at follow-up eliciting information on staff attitudes toward alcohol screening and brief intervention (SBI), previous practice of alcohol SBI, preparedness to undertake these activities, the training required to implement SBI, the suitability of each site to provide SBI [34]	Postal survey examining: perceived barriers or enablers of implementation of SBIs in Ireland
Effectiveness	Direct (interview at baseline and follow-up): AUDIT [38] Other drug use (eg, Maudsley Addiction Profile [39]) Motivation to change risky behavior (eg, Readiness ruler [40])		Indirect (review of clinical records): Results of chemical tests for alcohol and drugs (eg, breathalyzer or urine tests) will be also retrieved using the practice records to verify self-report measures

### Data Analysis

Descriptive statistics will be estimated with respect to key feasibility variables. At baseline, rates of practice and patient recruitment, prevalence of problem drug use at participating practices, and baseline prevalence of problem alcohol use among problem drug users will be estimated. Process and fidelity evaluation of pilot educational intervention will be explored. Practice/patient retention rates, prevalence of problem alcohol use among problem drug users, and confounding factors such as practice busyness or person who performed SBI will be analyzed for outcome measures.

SPSS v20 and R software will be used for analysis by the HRB Center for Support and Training in Analysis and Research.

### Qualitative Evaluation

A parallel qualitative evaluation will also be conducted with patients and health care professionals.

With regard to health care professionals, open-ended questions will be asked eliciting information on staff attitudes toward alcohol SBI, previous practice of alcohol SBI, preparedness to undertake these activities, the training required to implement SBI, the suitability of each site to provide SBI, and other barriers to effective implementation [34].

With regard to patients, among a 20% purposive sample (estimated N=16) of patients in the intervention practices, we will also explore patients’ satisfaction with and experience of intervention and care related to problem alcohol use in the preceding 3-6 months. Interviews will be done by researcher via telephone, postal questionnaire, or in person. Prior to the interviews, the participant will be informed of the interview purpose, the interview procedure, and the use of the findings. The participant will then be invited to sign an additional consent form and the interview will commence.

Qualitative data analysis will be systematic and organized to easily locate information within the dataset when tracing results, providing examples in context [43]. The qualitative research software Nvivo v8 will be used to facilitate the coding. Thematic analysis will be used to analyze qualitative data. This approach has many benefits for such an interpretive study, as it is a “method for identifying, analyzing, and reporting patterns (themes) within data” [43].

### Ethical Considerations

Ethical approval has been obtained from the Research Ethics Committee of the Irish College of General Practitioners (Protocol Reference: Cullen, 2012 Nov 29). Research carried out on humans in this study is in compliance with the Helsinki Declaration. The protocol follows the checklist of items to consider for inclusion in a report of a pilot studies [44], adopted from the CONSORT statement [45,46].

A two-stage procedure to obtain informed patient consent to participate in the study will be used during the study. Patients who consult a GP taking part in the study, and who in the clinical opinion of the GP are eligible for the study, will be given written information on the study (brief study information sheet). Those interested in participating will be invited to meet a researcher who will be at the practice during the recruitment period. At this meeting, interested patients will be given further information on the study and will have an opportunity to ask questions from the researcher. When all issues have been explained to patients’ satisfaction, they will be asked to indicate consent to participate in the study by signing a consent form and this procedure will be witnessed by a third party. The standard patient consent form for participation in nonclinical trials, developed by the Research Ethics Committee of the Irish College of General Practitioners, will be used in the study. Participation in the study will be on a voluntary basis. No inducements to participate will be offered to patients, and refusal to participate will not compromise patient care.

Potential adverse effects of the intervention will be explored in the qualitative interviews with patients and practitioners.

## Results

This study is ongoing and a protocol system is being developed for the study. This feasibility study may inform future research among the high-risk population of problem drug users and guide policy and service development locally and internationally by providing initial indications as to whether psychosocial interventions for problem alcohol use are feasible, acceptable, and also effective among problem drug users attending primary care.

## Discussion

The PINTA is the first study to examine the feasibility and acceptability of alcohol SBI for problem alcohol use among problem drug users attending primary care. It will provide key data that will enhance scientific understanding of interventions that prevent risk behaviors, inform policy and service development, and contribute to health and social gain locally and internationally.

The project team involves academic, clinical, policy experts responsible for planning/delivery of addiction care/primary care, and international experts on optimum primary care delivery to at-risk populations/primary care alcohol treatment.

The proposed work will build on our recently completed project that has identified problem alcohol use as a common finding among patients on methadone and subsequent program of research, which has explored and documented existing practices with respect to alcohol interventions among this group. This information is used, in conjunction with scientific evidence, to develop clinical guidelines regarding screening and treatment for problem alcohol use, and then consult it with patients and health care professionals.

At the end of this research, the feasibility of a clinical intervention, informed by international best practice and local barriers, will be evaluated in areas of high need. This intervention is likely to consist of a training and support program and clinical guidelines. By involving service users and service providers in their development phase, acceptability and feasibility will be enhanced. The research methodology also gives a voice to a group of service users not normally at the center of how interventions are tested.

This feasibility study may inform clinical practice by providing initial indications as to whether psychosocial interventions for problem alcohol use are feasible, acceptable, and also effective among problem drug users attending primary care. It will also inform future research on the topic by providing key parameters for the design of a future cluster RCT. This study is ongoing and a protocol system is being developed for the study.

## Abbreviations

AUDIT: Alcohol Use Disorders Identification Test
GP: general practitioner
HSE: Health Services Executive
MAP: Maudsley Addiction Profile
MRC: Medical Research Council
PINTA: psychosocial intervention for alcohol use among problem drug users
RCT: randomized controlled trial
SAAPPQ: Shortened Alcohol and Alcohol Problems Perception Questionnaire
SBI: screening and brief intervention
